# 1354. Safety of AZD7442 (Tixagevimab/Cilgavimab) for Prevention of COVID-19: 15-Month Final Analysis of the PROVENT Phase 3 Study

**DOI:** 10.1093/ofid/ofad500.1191

**Published:** 2023-11-27

**Authors:** Myron J Levin, Andrew Ustianowski, Stéphane De Wit, Rohini Beavon, Jesse Thissen, Seth Seegobin, Katie Streicher, Alexandre Kiazand, Mark T Esser

**Affiliations:** University of Colorado Denver School of Medicine, Aurora, Colorado; North Manchester General Hospital, Manchester, England, United Kingdom; CHU St-Pierre, Brussels, Brussels Hoofdstedelijk Gewest, Belgium; AstraZeneca, Cambridge, England, United Kingdom; AstraZeneca, Cambridge, England, United Kingdom; AstraZeneca, Cambridge, England, United Kingdom; AstraZeneca, Cambridge, England, United Kingdom; AstraZeneca, Cambridge, England, United Kingdom; AstraZeneca, Cambridge, England, United Kingdom

## Abstract

**Background:**

In the PROVENT phase 3 pre-exposure prophylaxis study, AZD7442 (tixagevimab/cilgavimab) reduced symptomatic COVID-19 by 76.7% vs placebo at primary analysis (*P*< 0.001; median follow-up: 83 days) and was well tolerated. Here, we report final safety from PROVENT at ∼15 months of follow-up.

**Methods:**

In PROVENT (NCT04625725), adults without prior SARS-CoV-2 infection or COVID-19 vaccination, with increased risk of inadequate response to vaccination and/or SARS-CoV-2 exposure, were randomized 2:1 to either a 300-mg intramuscular dose of AZD7442 or placebo. Results are reported from the February 22, 2023 final data cut-off. The primary safety endpoint was assessment of adverse events (AEs), serious adverse events (SAEs), medically attended AEs (MAAEs), and AEs of special interest (AESIs). A cardiovascular events adjudication committee independently reviewed and adjudicated 5 event types (cardiac ischemia, cardiovascular death, heart failure, stroke, and thrombotic events). Efficacy data have previously been reported.

**Results:**

Across both the AZD7442 and placebo groups, 3669 (69.8%) participants completed the study. Median follow-up was 456 days in the AZD7442 group and 455 days in the placebo group. AEs occurred in 58.2% and 58.0% of participants administered AZD7442 and placebo, respectively (**Table**). Most AEs were mild to moderate in severity; 256 (7.4%) and 125 (7.2%) of participants in the AZD7442 and placebo groups, respectively, reported an AE of grade 3 (severe) or higher. SAEs occurred in 6.2% and 5.6% of AZD7442 and placebo participants, MAAEs in 28.6% and 25.3%, respectively, and deaths in 0.6% and 0.6%, respectively. AESIs occurred in 3.0% and 2.5% of AZD7442 and placebo participants, respectively, including 0.5% and 0.4% with cardiovascular events categorized as AESIs. Of 88 (2.5%) and 39 (2.2%) participants in AZD7442 and placebo groups with cardiovascular events evaluated by an adjudication committee, 40 (1.2%) and 12 (0.7%) had positively adjudicated events.

**Figure:**
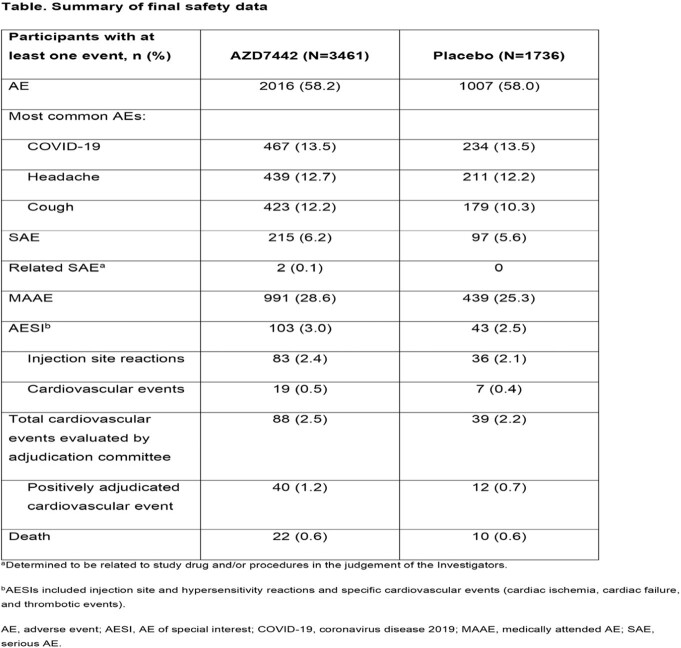

**Conclusion:**

This analysis provides further evidence to support the long-term safety of AZD7442 as prevention for COVID-19.

**Disclosures:**

**Myron J. Levin, MD**, Dynavax: Advisor/Consultant|GSK: Advisor/Consultant|GSK: Grant/Research Support|GSK: Data safety monitoring/Advisory board|Johnson & Johnson: Grant/Research Support|Merck & Co.: Advisor/Consultant|Moderna: Grant/Research Support|Novavax: Grant/Research Support|Pfizer: Advisor/Consultant|Seqirus: Advisor/Consultant **Andrew Ustianowski, MD, PhD**, Gilead: Honoraria|Gilead: Advisory Board|GSK: Honoraria|Janssen: Honoraria|Merck: Honoraria|Merck: Advisory Board|Sanofi: Honoraria|ViiV Healthcare/GSK: Advisory Board **Stéphane De Wit, MD**, AstraZeneca: Financial support for the conduct of this study **Rohini Beavon, PhD**, AstraZeneca: Employee|AstraZeneca: Stocks/Bonds **Jesse Thissen, MSc**, AstraZeneca: Employee|AstraZeneca: Stocks/Bonds **Seth Seegobin, PhD**, AstraZeneca: Employee|AstraZeneca: Stocks/Bonds **Katie Streicher, PhD**, AstraZeneca: Employee|AstraZeneca: Stocks/Bonds **Alexandre Kiazand, MD**, AstraZeneca: Employee|AstraZeneca: Stocks/Bonds **Mark T. Esser, PhD**, AstraZeneca: Employee|AstraZeneca: Stocks/Bonds

